# Correction: Imaging Findings of the Distal Radio-Ulnar Joint in Trauma

**DOI:** 10.5334/jbr-btr.966

**Published:** 2015-12-02

**Authors:** M. Mespreuve, F. Vanhoenacker, K. Verstraete

**Affiliations:** 1 St.-Maarten General Hospital, Leopoldstraat 2, 2800 Mechelen, Belgium; 2 University Hospital Ghent, Belgium; 3 University Hospital Antwerp, Belgium

**Keywords:** Wrist, injuries

## Abstract

This article details a correction to: Mespreuve, M, Vanhoenacker, F and Verstraete, K 2015 Imaging Findings of the Distal Radio-Ulnar Joint in Trauma. Journal of the Belgian Society of Radiology, 99(1), pp. 1–20, DOI: http://dx.doi.org/10.5334/jbr-btr.846

## Correction

**1. Fig. 2** on page 3: part of the caption (from C-D) should be replaced by:

C. The meniscus homologue (thick arrow) is interposed between the ulnar styloid and disc proximally and the cartilage of the triquetral bone distally.
D. The volar and dorsal part of the RUL (vertical arrows) are fixed between the radial notch and the base of the ulnar styloid. The m. extensor carpi ulnaris lies within his sulcus (horizontal arrow).
E. The m. extensor carpi ulnaris lies on the ulnar styloid (horizontal arrow). The central disc (TFC) (vertical arrows) is attached at the radial side to the cartilage of the radial sigmoïd notch and at the ulnar side to the styloid.

**2. Fig. 27** on page 14: the grey zone in the center of the figure 27 fails. Correct figure infra (as in the galley proof).

**Figure 27:**
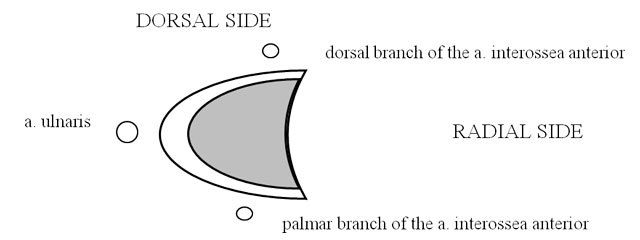
The correct image.

## References

[1] Mespreuve M, Vanhoenacker F, Verstraete K (2015). Imaging Findings of the Distal Radio-Ulnar Joint in Trauma. Journal of the Belgian Society of Radiology.

